# Automatic Prediction of Cardiovascular and Cerebrovascular Events Using Heart Rate Variability Analysis

**DOI:** 10.1371/journal.pone.0118504

**Published:** 2015-03-20

**Authors:** Paolo Melillo, Raffaele Izzo, Ada Orrico, Paolo Scala, Marcella Attanasio, Marco Mirra, Nicola De Luca, Leandro Pecchia

**Affiliations:** 1 Multidisciplinary Department of Medical, Surgical and Dental Sciences, Second University of Naples, Naples, Italy; 2 SHARE Project, Italian Ministry of Education, Scientific Research and University, Rome, Italy; 3 Department of Translational Medical Sciences, University of Naples Federico II, Naples, Italy; 4 School of Engineering, University of Warwick, Coventry, United Kingdom; Université de Montréal, CANADA

## Abstract

**Background:**

There is consensus that Heart Rate Variability is associated with the risk of vascular events. However, Heart Rate Variability predictive value for vascular events is not completely clear. The aim of this study is to develop novel predictive models based on data-mining algorithms to provide an automatic risk stratification tool for hypertensive patients.

**Methods:**

A database of 139 Holter recordings with clinical data of hypertensive patients followed up for at least 12 months were collected ad hoc. Subjects who experienced a vascular event (i.e., myocardial infarction, stroke, syncopal event) were considered as high-risk subjects. Several data-mining algorithms (such as support vector machine, tree-based classifier, artificial neural network) were used to develop automatic classifiers and their accuracy was tested by assessing the receiver-operator characteristics curve. Moreover, we tested the echographic parameters, which have been showed as powerful predictors of future vascular events.

**Results:**

The best predictive model was based on random forest and enabled to identify high-risk hypertensive patients with sensitivity and specificity rates of 71.4% and 87.8%, respectively. The Heart Rate Variability based classifier showed higher predictive values than the conventional echographic parameters, which are considered as significant cardiovascular risk factors.

**Conclusions:**

Combination of Heart Rate Variability measures, analyzed with data-mining algorithm, could be a reliable tool for identifying hypertensive patients at high risk to develop future vascular events.

## Introduction

Cardiovascular and cerebrovascular events (i.e., myocardial infarction, stroke) are the leading cause of premature death and disability in the developed countries[1–3]. Therefore, there has been great interest in the development of computational tools for prognosis and diagnosis of cardiac disease and, in particular, vascular events. The aim of these tools is to support cardiologists on prognostic and diagnostic tasks, reducing both the number of missed diagnoses or prognoses and reduce the time taken to reach such decisions. In literature, different risk factors for vascular events have been identified and are currently used for prognostics purposes, particularly, arterial intima media thickness (IMT), assessed by carotid ultrasound, and left ventricular mass, evaluated by echocardiography, have been proven as powerful predictor of future vascular events [4–7]. However, their positive predictive value should be constantly improved to comply with the higher possible quality level required for the clinical practice.

Heart rate variability (HRV) is a standard method for studying the control mechanisms of autonomic nervous system (ANS) on heart function and several studies showed that statistical, geometrical, spectral and nonlinear analysis of HRV are powerful tools for the evaluation of cardiovascular health and that HRV could be an independent risk factor for vascular events[8–10]. Sajadieh et al. showed that subjects with familial predisposition to premature heart attack and sudden death have reduced HRV[8]. Dekker et al. concluded that low HRV is associated with increased risk of coronary heart disease and death from several causes[9]. Binici et al. demonstrated that depressed nocturnal heart rate variability is a strong marker for the development of stroke in apparently healthy subject[10]. These previous studies focused on the most common linear HRV measures, suggesting that HRV could be useful for adoption in clinical practice.

Since HRV can be expressed using several measures, some recent studies proposed automatic classification and feature selection algorithms for diagnosis of cardiovascular diseases[11–16] or stressful conditions[17, 18]. The performance of these classifiers in prognostic or diagnostic tasks is relatively high (80% to 95% sensitivity in the best cases); however, they have been used for the recognition of several patterns in specific cardiac diseases (e.g., Congestive Heart Failure, paroxysmal atrial fibrillation, myocardial infarction, cardiac arrhythmias, amongst others) rather than for the prognosis of cardiovascular risk. Few studies focussed on automatic cardiovascular risk assessment based on HRV. Ramirez-Villegas et al. adopted HRV and pattern recognition techniques to discriminate between healthy control subjects and cardiovascular risk patients[19]. Singh and Guttag proposed classification tree-based risk stratification models to predict 90 day mortality in patients who suffered from a non-ST elevation acute coronary syndrome[20]. Recently, Song et al. developed Support Vector Machine (SVM) models to quantify the risk of cardiac death in patients after acute myocardial infarction[21], while Ebrahimzadeh et al. proposed a novel approach to distinguish between patients prone to Sudden Cardiac Death and normal people[22].

In the present study, linear and nonlinear HRV analysis methods and pattern recognition schemes were used to discriminate between cardiovascular high risk and low risk hypertensive patients. The risk of developing a vascular event was assessed over a one-year follow-up after electrocardiographic recordings. The developed classifier achieved high sensitivity and specificity rates in automatically identifying patients developing vascular events one year within electrocardiographic recording.

## Materials and Methods

### Dataset

The current study was performed on a database containing nominal 24-h electrocardiographic (ECG) holter recordings of 139 hypertensive patients aged 55 and over (including 49 female and 90 male, age 72 ± 7 years), recruited between 1 January 2012 to 10 November 2013 at the Centre of Hypertension of the University Hospital Federico II. The ECG Holter was performed after a one-month antihypertensive therapy wash-out. The patients were followed up for 12 months after the recordings in order to record major cardiovascular and cerebrovascular events, i.e. fatal or non-fatal acute coronary syndrome including myocardial infarctions, syncopal events, coronary revascularization, fatal or non-fatal stroke and transient ischemic attack. All the events were adjudicated by the Committee for Event Adjudication in the Hypertension Center. Adjudication was based on patient history, contact with the reference general practitioner and clinical records documenting the occurrence of the event/arrhythmia[23, 24]. Among the study sample, in the 12-month follow-up after recordings, 17 patients experienced a recorded event (11 myocardial infarctions, 3 strokes, 3 syncopal events) and for that reason, were considered as high-risk subjects, while the remaining ones as low-risk subjects. Moreover, the patients were evaluated by a cardiac and carotid ultrasonography. Left ventricular mass was determined by using the formula developed by Devereux[25] as recommended by American Society of Echocardiography (ASE)[26] and divided by the body surface area to calculate left ventricular mass index (LVMi, g/m^2^). B-mode ultrasonography of carotid arteries was performed in order to compute the maximum IMT (mm). Further details about the ECG recording, the cardioecographic and carotid ultrasonographic procedures can be found in a previous report[27]. The current study was approved by the Ethics Committee of Federico II University Hospital Trust and the data were collected by the Department of Translational Medical science of the University of Naples Federico II in the framework of the Smart Health and Artificial intelligence for Risk Estimation (SHARE) project. All the participants signed informed consent for the use of data for scientific purposes. The whole dataset could be downloaded as "Smart Health for Assessing the Risk of Events via ECG database" from the physionet.org website[28].

### HRV processing

The series of beat intervals (RR) were obtained from ECG recordings using an open-source software for QRS detection[29]. A stationary segment of 5 minutes recorded during daytime was randomly selected for each subject[15]. Stationarity was assessed by a stationarity test based on time-frequency features of the surrogates[30].

Standard linear HRV analysis according to International Guidelines was performed[31]. A number of standard time-domain HRV measures were calculated: Average of all RR intervals (AVNN), standard deviation of all RR intervals (SDNN), square root of the mean of the sum of the squares of differences between adjacent NN intervals (RMSSD), number and percentage of differences between adjacent RR intervals that are longer than 50 ms (NN50 and pNN50, respectively), HRV triangular index (HRVTi), i.e. the proportion of all accepted RR intervals to their modal measurement at a discrete scale of 1/128s bins, triangular interpolation of RR interval histogram (TI), i.e. the baseline width of the distribution measured as a base of a triangle, approximating the RR interval distribution by using the minimum square difference.

The frequency-domain HRV measures relied on the estimation of power spectral density (PSD) computed, in this work, with the Lomb-Scamble periodogram[32]. The generalized frequency bands in case of short-term HRV recordings are the very low frequency (VLF, 0–0.04 Hz), low frequency (LF, 0.04–0.15 Hz), and high frequency (HF, 0.15–0.4 Hz). The frequency-domain measures extracted from the PSD estimated for each frequency band included absolute and relative powers of VLF, LF, and HF bands, LF and HF band powers in normalized units, the LF/HF power ratio, and peak frequencies for each band. The relative powers and the peak frequencies were indicated with the suffices % and peak, respectively, for example LF_%_ and LF_peak_ indicated the LF power normalized to the Total Power (TP) and the peak frequency of LF, respectively.

Moreover, nonlinear properties of HRV were analysed by the following methods: Poincaré Plot (features SD_1_ and SD_2_)[11, 33], Approximate Entropy (AppEn)[34], Sample Entropy (SampEn)[35], Correlation Dimension (CD)[36], Detrended Fluctuation Analysis (features: Alpha_1_ and Alpha_2_)[37, 38], and Recurrence Plot [39–41]. Details about the non-linear measurements were reported in S1 Appendix. Further details about the methods could be found elsewhere[18, 42]. The HRV analysis was performed using an *ad hoc* developed HRV software based on MATLAB implementation[43].

### Statistical analysis, feature selection and data-mining methods

All values of continuous and categorical variables were presented as mean ± standard deviation and as count and percentage, respectively. Unpaired t-tests were adopted to compare continuous clinical variable, while chi-square tests were used to compare categorical variables between those who experienced a vascular event and those who did not.

In order to assess the generation ability of the models, we adopted the hold-out approach, i.e. the whole dataset was split into two subsets: training set (60% of instances) and test set (the remaining 40% of instances). The training set was used for feature selection and choice of the optimal parameters. The test set was adopted to evaluate the performance of the developed classifiers (with the features and parameters chosen on training set): ROC curves were constructed to compare the predictive value of each method for predicting vascular events and accuracy, sensitivity, specificity were computed according to standard formulae.

Since the number of HRV measures was high compared to the instances and some of them were strongly correlated, we adopted a chi-squared statistics[44] and a correlation-based [45] feature selection methods to filter out irrelevant and redundant features. The first method ranked the features by computing the value of the chi-squared statistic of each feature with respect to the classification problem. The second method scores the worth of subsets of features by taking into account the usefulness of individual features for predicting the class along with the level of intercorrelation among them with the belief that good feature subsets include features highly correlated with the class, yet uncorrelated with each other. Moreover, we computed the feature importance measures based on Random Forests (RF)[46].

Several data-mining approach were used to develop classifier for vascular event prediction based on HRV features, including Naïve Bayes classifier(NB), decision trees using the C4.5 decision tree induction algorithm, RF, boosting meta-learning approach i.e. AdaboostM1 (AB), SVM and artificial neural networks using a Multilayer Perceptron (MLP). More details about the algorithms and the optimal parameter choice could be found in S2 Appendix.

## Results

The clinical characteristics of the study sample of patients were reported in Table 1. No statistical differences were detected between the two groups of patients.

**Table 1:** Patient baseline characteristics.

Clinical Features	Low-risk subjects	High-risk subjects	p-value
Age (years)	71.4±7	74.1±6.5	0.136
Sex (female)	41 (33.6%)	8 (47.1%)	0.277
Family history of hypertension	41 (33.6%)	7 (41.2%)	0.622
Family history of stroke	10 (8.2%)	3 (17.6%)	0.236
Smoking	35 (28.7%)	5 (29.4%)	0.983
Diabetes	18 (14.8%)	3 (17.6%)	0.834
Diastolic Blood Pressure (mmHg)	76.3±9.1	73.5±8.4	0.204
Systolic Blood Pressure (mmHg)	136.6±19.5	141.7±23.5	0.326
Total Cholesterol (mg/dl)	175.7±35.1	182.9±42.7	0.460
Low Density Lipoprotein (mg/dl)	101±30.1	102±34.3	0.907
High Density Lipoprotein (mg/dl)	52.4±13.1	53.3±15.3	0.813
Body Mass Index (kg/m^2^)	27.6±3.9	27.9±4.9	0.793
Body Surface Area (m^2^)	1.9±0.2	1.9±0.2	0.442
Alpha-blockers	17 (13.9%)	3 (17.6%)	0.782
Beta-blockers	50 (41%)	6 (35.3%)	0.487
ACE inhibitor	37 (30.3%)	8 (47.1%)	0.247
Dihydropyridine	27 (22.1%)	7 (41.2%)	0.131
Intima Media Thickness (mm)	2.3±0.7	2.4±1.1	0.685
Left Ventricular Mass index (g/m^2^)	130.1±26.1	140.2±25.1	0.135
Ejection Fraction (%)	59.3±10.9	57.8±13	0.591

Data are expressed as mean and standard deviation for continuous variables (e.g. age) and as count and percentage of patients per each group for categorical variables (e.g. gender).

**Fig 1:**
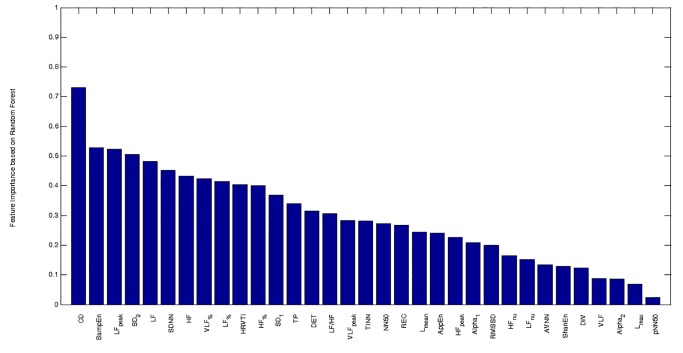
Feature importance computed by using Random Forest algorithm. CD: Correlation dimension. SampEn: Sample entropy. LF_peak_: peak frequency of LF band. SD_2_: long-term variability in Poincaré Plot. LF: absolute power in low frequency band (0.04–0.15 Hz). SDNN: standard deviation of all RR intervals. HF: absolute power in high frequency band (0.15–0.4 Hz). VLF_%_: relative power in very low frequency band (0–0.04 Hz). LF_%_: relative power in low frequency band (0.04–0.15 Hz). HRVTi: HRV triangular index. HF_%_: relative power in high frequency band (0.15–0.4 Hz). SD_1_: short-term variability in Poincaré Plot. TP: total power. DET: determinism. LF/HF: the ratio between LF and HF. VLF_peak_: peak frequency of VLF band. TINN: triangular interpolation of RR interval histogram. NN50: number of differences between adjacent RR intervals that are longer than 50 ms. REC: recurrence rate. L_mean_: mean length of lines in recurrence plot. AppEn: Approximate Entropy. HF_peak_: peak frequency of HF band. Alpha_1_: short-term fluctuations in Detrended Fluctuation Analysis. RMSSD: square root of the mean of the sum of the squares of differences between adjacent RR intervals. HF_nu_: power in high frequency band (0.15–0.4 Hz), expressed in normalized unit. LF_nu_: power in low frequency band (0.04–0.15 Hz), expressed in normalized unit. AVNN: Average of all RR intervals. ShanEn: Shannon Entropy. DIV: Divergence. VLF: absolute power in very low frequency band (0–0.04 Hz). Alpha_2_: long-term fluctuations in Detrended Fluctuation Analysis. L_max_: maximal length of lines in recurrence plot. pNN50: percentage of differences between adjacent RR intervals that are longer than 50 ms.

Among the 33 HRV features, the chi-squared statistics feature selection method identified as relevant the following features (reported in descending order of ranking): CD, SampEn, SD_2_, SDNN, LF, LF_peak_, HF, HRVTi, TP, LF_%_, while the correlation-based algorithm selected the subset of the following features: HRVTi, LF, HF, LF_%_, LF_peak_, SD_2_, SampEn, CD. Finally, Fig. 1 showed the importance of each feature as computed by the RF algorithm. All the features identified by the feature selection methods were ranked among the ten most important features by RF, with the only exception of TP, which was ranked as 13^rd^.

For each data-mining method, the optimal combination of parameters and the best subset of input features were selected by maximizing the accuracy estimated by 10-fold-crossvalidation as shown in Table 2. C4.5 and AB achieved the highest performances with chi-squared feature selection algorithm, while MLP and NB with the the correlation-based algorithm. SVM and RF performed well with all the features.

**Table 2:** Performance measurement (10-fold-crossvalidation estimation) of the proposed algorithms based on HRV features.

Classifier	Parameters	Feature selection (# features)	AUC	ACC	SEN	SPE
AB	NI: 220; CF 0.5; MI: 20	None (33)	94.5%	91.8%	93.2%	90.4%
AB	NI: 20; CF: 0.3; MI: 10	CFS (8)	92.2%	85.6%	86.3%	84.9%
**AB**	**NI: 120; CF: 0.45; MI: 10**	**Χ** ^**2**^-**FS(10)**	**94.7%**	**89.0%**	**90.4%**	**87.7%**
C4.5	CF: 0.3; MI: 5	None (33)	80.3%	76.7%	78.1%	75.3%
C4.5	CF: 0.3; MI: 5	CFS (8)	82.8%	80.8%	87.7%	74.0%
**C4.5**	**CF: 0.1; MI: 5**	**Χ** ^**2**^-**FS (10)**	**83.0%**	**76.7%**	**76.7%**	**76.7%**
MLP	LR 0.3; M 0.6; NE 200	None (33)	86.7%	82.9%	80.8%	84.9%
**MLP**	**LR 0.6; M 0.4; NE 200**	**CFS (8)**	**86.9%**	**78.1%**	**86.3%**	**69.9%**
MLP	LR 0.3; M 0.2; NE 1800	Χ^2^-FS (10)	86.1%	78.8%	82.2%	75.3%
NF	-	None (33)	72.4%	65.8%	76.7%	54.8%
**NF**	-	**CFS (8)**	**80.1%**	**70.5%**	**78.1%**	**63.0%**
NF	-	Χ^2^-FS (10)	77.8%	71.9%	82.2%	61.6%
**RF**	**NT 300 NF 5**	**None (33)**	**94.5%**	**88.4%**	**91.8%**	**84.9%**
RF	NT 20 NF 5	CFS (8)	92.3%	87.7%	90.4%	84.9%
RF	NT 400 NF 4	Χ^2^-FS (10)	93.2%	89.0%	93.2%	84.9%
**SVM**	**G: 1.4**	**None (33)**	**93.1%**	**89.0%**	**86.3%**	**91.8%**
SVM	G: 2.3	CFS (8)	89.1%	81.5%	84.9%	78.1%
SVM	G: 1.6	Χ^2^-FS (10)	89.2%	80.8%	86.3%	75.3%

CFS: correlation-based feature selection algorithm (a subset of 8 HRV features)

Χ^2^-FS: chi-squared feature selection algorithm (a subset of 10 HRV features)

NI: number of iteration

ML: minimum number of instances per leaf.

CF: confidence factor for pruning

LR: learning rate

M: momentum

NE: number of epoch

NT: number of trees

NF: number of randomly chosen features

G: gamma

AUC: area under the curve

CI: confidence interval

ACC: accuracy

SEN: sensitivity

SPE: specificity

In bold: the best performances of each classifier.

The performance measurements estimated on the independent test set are reported in Table 3 for each classification algorithm based on HRV features. The RF outperformed the other data-mining methods by achieving the best value of performance measures, i.e., an accuracy of 85.7%, a sensitivity of 71.4%, and a specificity of 87.8%. The prediction based on the echographic parameters, i.e., IMT and LVMi, resulted in a very low sensitivity rate (<45%), as shown in Table 4.

**Table 3:** Performance measurements estimated on the test set (hold-out estimation) of the best classifiers based on HRV features.

Class.	Parameters	Feature selection (# features)	AUC	ACC (95% CI)	SEN	SPE
AB	NI: 120; CF: 0.45; MI: 10	Χ^2^-FS(10)	81.9%	83.9%(76.9–86.6)	71.4%	85.7%
C4.5	CF: 0.1; MI: 5	Χ^2^-FS (10)	69.8%	75.0% (67.7–79.1)	57.1%	77.6%
MLP	LR: 0.6; M: 0.4; NE: 200	CFS (8)	64.7%	76.8% (69.5–80.6)	42.9%	81.6%
NF	-	CFS (8)	74.9%	69.6% (62.4–74.4)	57.1%	71.4%
RF	NT: 300 NF: 5	None (33)	88.8%	85.7% (78.7–88.1)	71.4%	87.8%
SVM	G: 1.4	None (33)	90.1%	83.9% (76.9–86.6)	71.4%	85.7%

Class.: Classifier

AB: Adaboost

MLP: Multilayer Perceptron

NB: Naïve Bayes classifier

RF: Random Forest

SVM: Support Vector Machine

NI: number of iteration

ML: minimum number of instances per leaf.

CF: confidence factor for pruning

LR: learning rate

M: momentum

NE: number of epoch

NT: number of trees

NF: number of randomly chosen features

G: gamma

Χ^2^-FS: chi squared feature selection algorithm (a subset of 10 HRV features)

CFS: correlation-based feature selection algorithm (a subset of 8 HRV features)

AUC: area under the curve

ACC: accuracy

CI: confidence interval

SEN: sensitivity

SPE: specificity.

**Table 4:** Performance measurements of classification based on echographic parameters.

Parameter	AUC	ACC (95% CI)	SEN	SPE
LVMi	63.5%	69.5% (69.9–73.0)	41.2%	73.9%
IMT MAX	49.1%	61.9% (57.3–65.8)	40.0%	64.9%

LVMi.: Left ventricular mass index

IMT MAX: maximum of intima media thickness

AUC: area under the curve

ACC: accuracy

CI: confidence interval

SEN: sensitivity

SPE: specificity.

**Fig 2:**
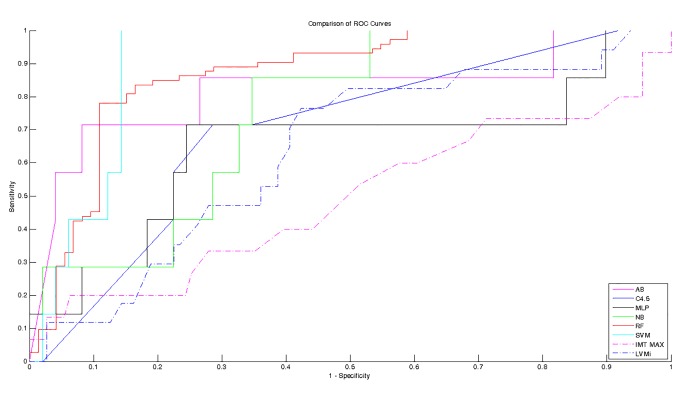
Receiver-operator characteristic curves for predicting vascular events by HRV-based classifiers and echographic parameters. The HRV-based classifiers are able to predict vascular events with higher sensitivity and specificity rate than echographic parameters. Sensitivity is determined from the proportion of patient developing a vascular event identified as high risk; specificity is determined from the proportion of patient free of vascular events identified as low risk. Solid lines represent classifier based on HRV features, dash-dot lines represent classifications based on echographic parameters. AB: Adaboost. MLP: Multilayer Perceptron. NB: Naïve Bayes classifier. RF: Random Forest. SVM: Support Vector Machine. LVMi.: Left ventricular mass index. IMT MAX: maximum of intima media thickness.

The ROC curves (estimated on the independent test set) for predicting vascular events over twelve months with HRV or echographic parameters are compared in Fig. 2. The HRV-based classifier showed higher AUC compared to echographic parameters. Among clinical parameters, the higher AUC was achieved by LVMi, followed by IMT. The other clinical available parameters (e.g. blood pressure, cholesterol) resulted in ROC with AUC lower than 0.5, i.e., worst performance than random choice, and for that reason, they are omitted. Among HRV-based classifier, SVM achieved the highest AUC, followed by RF.

**Fig 3:**
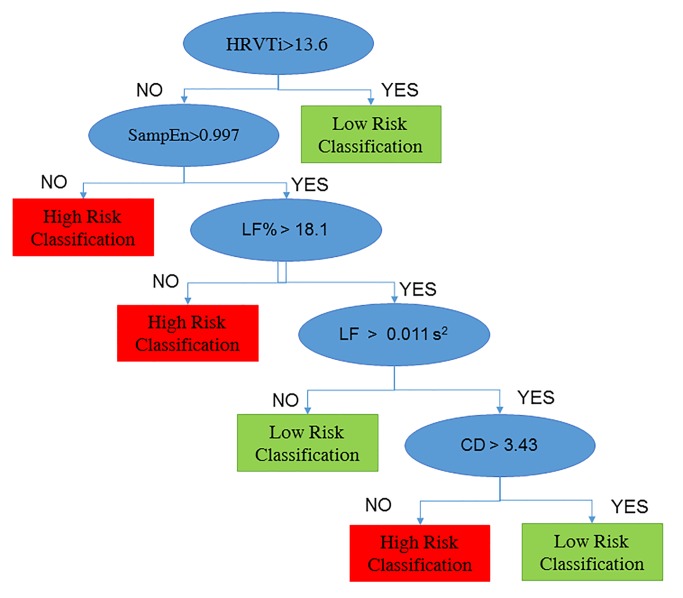
Decision tree for prediction of vascular events. The decision tree shows the set of rules adopted for classify high and low risk subjects: if HRVTi is higher than 13.6, the subject is classified as low risk, otherwise if SampEn lower than 0.997 or LF% lower than 18.1%, the subject is classified as high risk. The remaining subjects (with higher SampEn and LF_%_), are classified based on LF and CF: as high risk, if LF is higher than 0.001 s^2^ and CD is lower 3.43, otherwise as low risk. HRVTi: HRV Triangular Index. SampEn: Sample Entropy. LF: Low Frequency. LF_%_: Low Frequency expressed as percentage of Total Power. CD: correlation dimension.

Since AB achieved satisfactory performances, it was interesting to observe the rules obtained from the decision tree with the highest weight, shown in Fig. 3:

- the subject was classified as low-risk if HRVTi>13.6;
- a depression of HRVTi (<13.6) associated with a decreased SampEn (<0.997) or decreased LF_%_ (<18.1%) leaded to high-risk classification;
- otherwise, the subject was classified based on LF and CD, in particular, reduced CD (<3.43), although with LF > 0.011 s^2^, leaded to high-risk classification, otherwise, the subject was classified as low-risk.

## Discussion

In this study, we used HRV features extracted from 5 minutes excerpts of 24-hour clinical electrocardiographic dataset from hypertensive patients to develop a computer-aided predictive tool that improves risk stratification. Tree-based models applied on HRV features resulted effective in identifying high-risk patients among a population of hypertensive patients.

Linear HRV features demonstrated prognostic value for vascular events[8–10]. Nevertheless, these traditional measures had only a partial predictive capability. In this study, to advance the predictability of vascular events in hypertensive patients over twelve months, several data-miming approach were tested by combining linear and non-linear HRV features. The feature selection and ranking showed that nonlinear features, particularly CD, SampEn and SD_2_, increased the discrimination power when they were used in combination with the linear HRV features, such as HRVTi, LF, and HF. As a result, we proposed tree-based models, which resulted to be effective at predicting vascular events among hypertensive. Nevertheless, our results clearly showed that the HRV-based classifiers had a better prognostic capacity compared with LVMi and IMT, which are considered as powerful predictors of vascular events[4–6].

The sensitivity and specificity rates obtained in the current study were comparable with the performances achieved by Ebrahuimzaded et al.[22] and by Song et al.[21], who recently proposed HRV-based classifier for prediction of sudden cardiac death. However, in the present study none of the cardiovascular and cerebrovascular events occurred over the follow-up was fatal. Moreover, in the current study, we adopted a nested cross-validation approach: an inner 10-fold-crossvalidation loop was performed for model section (i.e., features selection and machine learning parameter optimization), while a hold-out test set was used to obtain almost unbiased estimates of the true classification performances.

The sets of rules of the tree models presented were consistent with the findings of previous studies, even if no medical a priori knowledge was adopted in the data-mining methods. In fact, depressed HRV was showed to be associated with high cardiovascular risk in previous studies[8–10]. Since HRV was proven to be the result of changes in heart rate caused by fluctuations in sympathetic and parasympathetic outflow (the two branches of ANS), less compensatory change, as evaluated by depressed HRV, suggested a less adaptive ANS. One of the reasons could be that ANS resulted less sensitive for minor hemodynamic changes in some hypertensive patients, which could have been a direct cause of the vascular event registered in this study. Furthermore, a possible mechanism underlying our findings could be low-grade inflammation: it has been suggested that autonomic imbalance could activate inflammation by influencing the bone marrow and lymphoreticular system and increased inflammation is associated with higher risk of cardiovascular events[47]. Finally, another possible explanation for the association between HRV and vascular risk was that individuals with low HRV already suffered from subclinical or silent vascular disease, which, if not detected, resulted in cardiovascular events in the following months[48].

As regards the comparison of data-mining methods, RF showed extremely good performance in the current study when comparing several methods for diagnosis of congestive heart failure based on HRV features, confirming previous findings[16]. Moreover, RF and SVM performed well without any feature selection, consistently with the capability of these algorithms to constitute embedded feature selection strategy, as demonstrated in previous studies[49, 50].

The clinical feasibility and uptake of the developed tool are now tested in a prospective study in subjects aged 55 and over recruited by the Center of Hypertension of the University Hospital of Naples. The physicians accessed the tool by an *ad hoc* developed web-based application; they could upload the ECG signals by a Windows application, a browser or an Android App. More details about the developed platform were reported elsewhere[51]. The physicians can visualize the signals, the HRV features and the results of the tool by using a web browser. The involved clinicians are pleased to use the tool and confirmed that it is clinical feasible and could be useful in clinical practice. They have specialist background in cardiology or emergency medicine and experience with ECG Holter analysis. Moreover, since 5-minute HRV measurement is inexpensive, easy to assess, and non-invasive, future research will focus on the clinical applicability of the system as a screening tool in non-specialized ambulatories (e.g. at General Practitioners’), in order to identify high-risk patients to be shortlisted for more complex (and costly) investigations. Improved identification of individuals at risk for the development of vascular events may result in more targeted and adequate prevention strategies.

The current study had the following limitations. First, we used only linear and nonlinear HRV features and not strong risk markers, such as Heart Rate Turbulence or T wave alterations. Secondly, further investigations are needed to assess whether the proposed models can perform well using other datasets, since the dataset of the current study was relatively small and unbalanced. Therefore, this novel predictive approach should be studied in a larger number of patients.

## Conclusions

This study proposed an automated system for prediction of vascular events in the following year using HRV analysis. The developed classifier enabled to identify hypertensive patients, which will undergo a cardiovascular event or stroke many weeks/months before the events by using a 5-minute ECG recording, achieving sensitivity and specificity rates of 71.4% and 87.8%.

Finally, since some echographic parameters have been proven as power predictors of vascular events[4–6], we compared the performance of our classifier with decision rules based on these parameters and we showed that the HRV-based system outperformed the classification based on echographic parameters. These findings confirmed that HRV could be a good predictor of future vascular events in the following year among hypertensive patients.

## Supporting Information

S1 Appendix Nonlinear HRV measurements. (DOCX)

S2 Appendix Data-mining methods. (DOCX)
