# Supplementary material for: Automatic Prediction of Cardiovascular and Cerebrovascular Events Using Heart Rate Variability Analysis
Source: PLoS One. 2015 Mar 20;10(3):e0118504. doi: 10.1371/journal.pone.0118504 (PMC4368686; doi:10.1371/journal.pone.0118504)
Supplement: S1 Appendix — (DOCX) [file pone.0118504.s001.docx]

# **S1 Appendix. Nonlinear HRV measurements**

Poincaré plot is a scatter plot of successive RR intervals that produces two standard deviation measures, SD_1_ describing short-term variability and SD_2_ describing long-term variability analysis[1].

ApEn measures the complexity or irregularity of the RR series[2]. It is a statistical measure used to quantify the regularities in data without a priori knowledge of the problem. SampEn is a similar but less biased measure than the ApEn to evaluate the regularity of time-series[3]. CD is also a measure of signal complexity and is expected to give information on the minimum number of dynamic variables needed to model the underlying system[4]. According to the recommendation for slow dynamic time series, such as heart rate variability, [5, 6] the embedding dimension was 2 and the tolerance 0.2 times SDNN in ApEn and SampEn computation, whereas in CD computation the embedding dimension was 10.

Detrended Fluctuation Analysis (DFA) measures the correlations within the signal at different time scales, divided into short-term fluctuations (Alpha_1_, within range 4–16 beats) and long-term fluctuations (Alpha_2_, within range 16–64 beats). [7, 8]

The recurrence plot is a technique that projects a time series in a higher dimensional space, called phase space. In that space, the pairwise Euclidean distance between all points is computed, creating a matrix where each row and each column is a point in the phase space, and each element of the matrix is the respective distance. When this distance is smaller than a given threshold *r*, that is two points are close in the phase space, a ‘recurrence’ occurs. In this study, according to [6, 9], the following values of the parameters introduced above were chosen:$m=10; r=\sqrt{m}*SDNN$. In the RP, lines are defined as series of diagonally adjacent recurrences. The length *l* of a line is the number of points which the line consists of. The following measures of RP were computed: recurrence rate *(REC)* defined in equation 1; maximal length of lines (*l_max_*); mean length of lines (*l_mean_*); the determinism (*DET*) defined in equation 2; the Shannon Entropy (*ShEn*) defined in equation 3.

(1) $REC=\frac{1}{K^{2}}\sum_{i=1}^{K} \sum_{j=1}^{K} M_{2}(i,j)$

(2)$DET= \frac{\sum_{l=2}^{l_{\max}} l *N_{l}}{\sum_{i=1}^{K} \sum_{j=1}^{K} M_{2}(i,j)}$, with N*_l_* = number of lines of length *l*

(3)$ShEn=\sum_{l=l_{\min}}^{l_{\max}} n_{l}*\ln n_{l}$, with n*_l_* = percentage of N*_l_* over all the number of lines.

1. Brennan M, Palaniswami M and Kamen P (2001) Do existing measures of Poincare plot geometry reflect nonlinear features of heart rate variability? IEEE Trans Bio Med Eng 48: 1342-1347.

2. Richman JS and Moorman JR (2000) Physiological time-series analysis using approximate entropy and sample entropy. American Journal of Physiology-Heart and Circulatory Physiology 278: H2039-H2049.

3. Lake DE, Richman JS, Griffin MP and Moorman JR (2002) Sample entropy analysis of neonatal heart rate variability. American Journal of Physiology-Regulatory, Integrative and Comparative Physiology 283: R789-R797.

4. Carvajal R, Wessel N, Vallverdú M, Caminal P and Voss A (2005) Correlation dimension analysis of heart rate variability in patients with dilated cardiomyopathy. Computer Methods and Programs in Biomedicine 78: 133-140.

5. Pincus SM (1991) Approximate Entropy as a Measure of System-Complexity. Proceedings of the National Academy of Sciences of the United States of America 88: 2297-2301.

6. Niskanen J-P, Tarvainen MP, Ranta-aho PO and Karjalainen PA (2004) Software for advanced HRV analysis. Computer Methods and Programs in Biomedicine 76: 73-81.

7. Penzel T, Kantelhardt JW, Grote L, Peter JH and Bunde A (2003) Comparison of detrended fluctuation analysis and spectral analysis for heart rate variability in sleep and sleep apnea. IEEE Trans Bio Med Eng 50: 1143-1151.

8. Peng CK, Havlin S, Stanley HE and Goldberger AL (1995) Quantification of Scaling Exponents and Crossover Phenomena in Nonstationary Heartbeat Time-Series. Chaos 5: 82-87.

9. Dabire H, Mestivier D, Jarnet J, Safar ME and Chau NP (1998) Quantification of sympathetic and parasympathetic tones by nonlinear indexes in normotensive rats. American Journal of Physiology-Heart and Circulatory Physiology 275: H1290-H1297.
