# Supplementary material for: Automatic Prediction of Cardiovascular and Cerebrovascular Events Using Heart Rate Variability Analysis
Source: PLoS One. 2015 Mar 20;10(3):e0118504. doi: 10.1371/journal.pone.0118504 (PMC4368686; doi:10.1371/journal.pone.0118504)
Supplement: S2 Appendix — (DOCX) [file pone.0118504.s002.docx]

# **S2 Appendix. Data-mining methods.**

NB uses the naive Bayes formula to calculate the probability of each class given the values of all the attributes and assuming the conditional independence of the attributes(1). A new instance is classified into the class with maximum calculated probability.

C4.5 is the landmark decision tree algorithm developed by Quinlan et al.(2). The feature of each node is selected in order to divide input samples effectively and information gain is used as a measure of effectiveness. After the induction of the decision tree, a pruning method was applied to reduce the tree's size and complexity.

RF is a state-of-the-art classifier developed by Breiman(3). It is composed of a number of decision trees that choose their splitting attributes from a random subset of *k* attributes at each internal node. The best split is taken among these randomly chosen attributes and the trees are built without pruning, as opposed to C4.5. One of the most relevant downsides of using RF, particularly in medical domain data-mining, is that its model is not easily understandable as a single tree.

AB is a meta-learning algorithm which works by incrementally running classifiers on samples of data instances and combining them into an aggregate model.(4) Each individual or weak classifier contributes to the aggregate model in proportion to its accuracy. After each iteration, data instances are reweighted based on incorrect aggregate classifications. This boosts the emphasis of misclassified instances, refining the construction of weak classifiers in future iterations. In the current study, C4.5 was adopted as weak classifier in the AB algorithm.

SVM belong to a general field of kernel-based machine learning methods and are used to efficiently classify both linearly separable and linearly inseparable data.(5) When the data are not linearly separable, they could be transformed to a higher dimensional space by using a transformation function, which is the so-called kernel function.

Multilayer perceptron (MLP) is one of the most popular neural network models due to its clear architecture and the simplicity of the algorithm(6). It consists of a network of nodes (processing elements) arranged in layers. The principle of the network is that when data are presented at the input layer, the network nodes perform calculations in the successive layers until an output value is obtained at each of the output nodes. This output signal should be able to indicate the appropriate class for the input data.

The choice of the algorithm parameters and best subset of features was based on the performances (i.e. accuracy, then sensitivity and finally specificity) estimated by 10-fold cross-validation. In the 10-fold cross-validation method, subjects are partitioned into two subsets in each round (totally 10 rounds): one with 90% subjects for training and the other with 10% subjects for validation. Repeating the test 10 times, the classification performance were then calculated by averaging the values obtained from the 10 validation subsets.

AB classifiers were developed by varying the number of iteration from 20 to 400 and C4.5 trees (both as single classifier and as base classifier in AB) were developed by varying confidence factor for pruning from 0.05 to 0.5, minimum number of instances per leaf from 5 to 20. MLP were trained by varying the learning rate from 0.3 to 0.9, the momentum from 0.2 to 1 and the number of epoch form 100 to 2000. RF was constructed using an ensemble of random trees from 20 to 400 with no depth limit and varying the number of randomly chosen features from log_2_(*n*)+1 to *n*, where *n* is the number of feature. As regards SVM, we used radial basis function kernel, varying gamma from 10^-5^ to 10.

We adopted a standard approach for learning from small and unbalanced dataset, based on oversampling that is the Synthetic Minority Over-sampling Technique (SMOTE)(7).

1. John GH, Langley P, editors. Estimating continuous distributions in Bayesian classifiers. Proceedings of the Eleventh conference on Uncertainty in artificial intelligence; 1995: Morgan Kaufmann Publishers Inc.

2. Quinlan JR. C4.5 : programs for machine learning. San Mateo, Calif.: Morgan Kaufmann Publishers; 1993.

3. Breiman L. Random Forests. Mach Learn. 2001;45(1):5-32.

4. Freund Y, Schapire RE, editors. Experiments with a new boosting algorithm. ICML; 1996.

5. Vapnik VN. Statistical learning theory. New York: Wiley; 1998. 736 p.

6. Bishop CM. Neural networks for pattern recognition. Oxford: Clarendon Press ; Oxford University Press; 1995. 482 p.

7. Chawla NV, Bowyer KW, Hall LO, Kegelmeyer WP. SMOTE: synthetic minority over-sampling technique. J Artif Intell Res. 2002;16(1):321-57.
